# FoxO1 Overexpression Ameliorates TNF-*α*-Induced Oxidative Damage and Promotes Osteogenesis of Human Periodontal Ligament Stem Cells via Antioxidant Defense Activation

**DOI:** 10.1155/2019/2120453

**Published:** 2019-10-31

**Authors:** Xiaojun Huang, Huan Chen, Yunyi Xie, Zeyuan Cao, Xuefeng Lin, Yan Wang

**Affiliations:** Guanghua School of Stomatology, Hospital of Stomatology, Sun Yat-sen University, Guangdong Provincial Key Laboratory of Stomatology, Guangzhou 510055, China

## Abstract

Periodontitis is a chronic disease that includes the pathologic loss of periodontal tissue and alveolar bone. The inflammatory environment in periodontitis impairs the osteogenic differentiation potential and depresses the regeneration capacity of human periodontal ligament stem cells (hPDLSCs). Since Forkhead box protein O1 (FoxO1) plays an important role in redox balance and bone formation, we investigated the role of FoxO1 in oxidative stress resistance and osteogenic differentiation in an inflammatory environment by overexpressing FoxO1 in hPDLSCs. First, we found that FoxO1 overexpression reduced reactive oxygen species (ROS) accumulation, decreased malondialdehyde (MDA) levels, and elevated antioxidant potential under oxidative condition. Next, the overexpression of FoxO1 protected hPDLSCs against oxidative damage, which involved stabilization of the mitochondrial membrane potential. Third, overexpressed FoxO1 promoted extracellular matrix (ECM) mineralization and increased the expression of the osteogenic markers Runx2 and SP7 in the inflammatory environment. These results indicated that FoxO1 overexpression in hPDLSCs has an anti-inflammatory effect, increases antioxidative capacity, and positively regulates osteogenesis in a mimicked inflammatory environment.

## 1. Introduction

Periodontitis, an infectious and chronic disease, mainly causes periodontium destruction and eventual tooth loss. Pathogens, including periodontopathic bacteria and viruses, activate lymphocytes, leading to an inflammatory response and oxidative stress [1]. Recently, increasing concern on the role of oxidative stress in the progression of periodontitis has been raised. Excessive reactive oxygen species (ROS) production from neutrophils and T/B lymphocytes results in lipid peroxidation, protein damage, and apoptosis. In addition, ROS mediate the NF-*κ*B signaling pathway and JNK signaling to enhance the expression of proinflammatory cytokines [2]. The secretion of proinflammatory factors, including TNF-*α*, IL-9, and IL-22, induces oxidative stress, matrix metalloproteinase formation, and osteoclast activation to accelerate periodontium loss and bone resorption [3]. To date, treatments for periodontitis, such as scaling, laser treatment, guided tissue regeneration, and other periodontal surgery, are efficacious against periodontal pathogens to an extent and increase periodontal attachment. However, these treatments still have limitations in the restoration of the periodontium to its natural shape and function.

Stem cell-based tissue engineering is expected to be a new therapeutic approach in periodontitis therapy to promote tissue regeneration and restore periodontal function [4, 5]. Human periodontal ligament stem cells (hPDLSCs) are a promising source of periodontal regeneration due to their self-renewal properties, multidifferentiation ability, biocompatibility, and immunomodulatory properties [4, 6]. Previous reports have demonstrated that hPDLSCs implanted with scaffold materials can repair alveolar bone defects in animal models [7]. However, the inflammatory environment in periodontitis is adverse to the desired tissue regeneration by hPDLSCs. Clinical examination found that patients with chronic periodontitis exhibit high malondialdehyde (MDA) levels and low antioxidant capacity in their saliva, indicating that antioxidative capacity is reduced during periodontitis progression [8, 9]. In addition, hPDLSCs from periodontitis patients exhibit impaired osteogenic capacity, including decreased expression of osteoblast markers and ALP activity and calcified nodule deposits [10]. Oxidative conditions impair the function of hPDLSCs and suppress their osteogenic differentiation potential, which can severely compromise periodontium regeneration and periodontitis therapy. Therefore, elevating the antioxidative and osteogenic differentiation capacity of hPDLSCs to resist the inflammatory environment in periodontitis is the key to achieving satisfactory therapeutic outcomes.

Forkhead box protein O1 (FoxO1), a member of the FoxO family, mediates glucose metabolism, tumorigenesis, oxidative stress, and bone formation [11, 12]. FoxO1 is involved in the recruitment of dendritic cells and the activation of adaptive immunity in periodontitis. Deletion of FoxO1 in dendritic cells led to a reduction in the immune response and the compensatory production of inflammatory cytokines. Consequently, the upregulation of inflammatory mediators caused alveolar bone resorption and attachment loss [13]. In addition, FoxO1 plays a crucial role in oxidant defense and protein synthesis in osteoblasts to control bone mass [12, 14]. In particular, the effect of FoxO1 on antioxidant defense is associated with bone formation. Mice with osteoblasts lacking FoxO1 showed increased oxidative stress, decreased osteoblast numbers, and low bone volume, but these effects were relieved by the antioxidant N-acetyl L-cysteine (NAC). Moreover, FoxO1 contributes to osteoblast proliferation and bone formation through its interaction with ATF4 to promote protein synthesis and regulation of the canonical Wnt pathway [12, 15]. However, whether FoxO1 contributes to antioxidant defense and osteogenesis to promote tissue regeneration in periodontitis remains unclear. This study is aimed at determining whether FoxO1 plays a protective role in hPDLSCs in the inflammatory microenvironment by elevating their antioxidative capacity and osteogenic differentiation.

## 2. Materials and Methods

### 2.1. Cell Isolation and Culture

The Ethics Committee of the Affiliated Stomatological Hospital of Sun Yat-sen University approved this research (ERC-[2016]-46). hPDLSCs were isolated from the periodontal ligaments of healthy human premolars extracted for orthodontic purposes. The ten healthy donors comprised 5 males and 5 females between 16 and 20 years of age. First, the periodontium was separated from the middle third of the extracted tooth root, and the periodontal ligament was digested in a solution containing 3 mg/mL collagenase (Life Technologies, USA) and 4 mg/mL dispase (Life Technologies) for 1 h at 37°C. Following termination of the enzyme reaction, cells were cultured in complete *α*-modified Eagle medium (*α*-MEM, Gibco, USA) containing 10% fetal bovine serum (FBS, Gibco, Grand Island), 100 U/mL penicillin, and 100 *μ*g/mL streptomycin (HyClone, Logan, UT, USA) at 37°C in 5% CO_2_.

### 2.2. Plasmid Construction and Establishment of Stable FoxO1-Overexpressing Cell Lines

The insert DNA (FoxO1 DNA fragment) was amplified with primers (5′-GCCGGAATTAGATCTCTCGAGAGTTAAGTTCTGGGCTCGC-3′ and 5′-CTCCCCTACCCGGTAGAATTCGTAACCTGCTCACTAACCCT-3′) using a cDNA template from hPDLSCs. The pMSCV-puro vector was digested with XhoI and EcoRI (TaKaRa, Japan). Both the PCR product and the linearized vector were purified by a Gel Extraction Kit (Omega, China), and the PCR product was inserted into the vector with a ClonExpress II One Step Cloning Kit (Vazyme, China) to create the pMSCV-FoxO1 plasmid. 5 *μ*g of the pMSCV-FoxO1 expression vector and 5 *μ*g of the PIK packaging vector were used to directly transfect 293T cells using the calcium phosphate method to generate lentiviral particles. An empty vector without the FoxO1 transgene was transfected into 293T cells as a control. At 48 h post transfection, the supernatant containing the lentiviral particles was harvested and centrifuged at 8000 rpm for 10 minutes at 4°C to clear cellular debris. hPDLSCs were infected with a lentiviral particle solution containing 8 *μ*g/mL polybrene and centrifuged at 2500 rpm for 1 h. Then, the virus­containing medium was replaced with fresh medium at 6 h lentiviral infection. At 48 h post infection, cells were selected with puromycin­containing medium. Stable FoxO1 overexpression cell line was named as PDLSC-pMSCV-FoxO1, and control cells were named as PDLSC-pMSCV-empty.

### 2.3. ROS Generation

2′,7′-Dichlorodihydrofluorescein diacetate (DCFH-DA) can diffuse into cells, where it is hydrolyzed by intracellular esterase to DCFH. Intracellular ROS oxidize nonfluorescent DCFH into fluorescent DCF. Intracellular ROS in hPDLSCs were measured by a Reactive Oxygen Species Assay Kit containing a DCFH-DA probe (Beyotime, China). Cells treated with 0 or 10 ng/mL TNF-*α* were harvested and incubated with DCFH-DA at 25°C for 20 min. The fluorescence intensity was analyzed by flow cytometry (Beckman, CytoFLEX) and fluorescence microscopy (ZEISS, Axio Observer Z1).

### 2.4. MDA Level Detection

MDA is the product of lipid peroxidation. Trichloroacetic acid (TBA) reacts with MDA to form a red TBA-MDA adduct that can be detected at 530-540 nm by spectrophotometry. The level of MDA in hPDLSCs following 0 or 10 ng/mL TNF-*α* treatment was detected by a Lipid Peroxidation MDA Assay Kit (Beyotime) according to the manufacturer's instructions. Cells were collected and lysed in lysis buffer to obtain the supernatant. Freshly prepared working reagent containing TBA was added to the supernatant, which was then incubated at 100°C for 15 min. The absorbance at 532 nm was measured on a spectrophotometer.

### 2.5. Western Blot Analysis

Cells were lysed in RIPA buffer at 4°C for 30 min. Protein concentrations in the hPDLSCs were measured by a Pierce BCA Protein Assay Kit (Thermo Scientific, Pierce, Germany). A total of 40 *μ*g of total protein from each sample was loaded onto an SDS-PAGE gel, and the proteins were separated by electrophoresis before being transferred onto PVDF membranes. After blocking in 5% nonfat milk, the membranes were incubated with primary antibody at 4°C overnight. Then, the membranes were washed three times with TBST buffer and incubated with HRP-linked antibody for 1 h at room temperature, followed by detection using the GeneGnome XRQ chemiluminescence imaging system. Primary antibodies against the following were used in this study: Runx2 (ABclonal, USA), SP7 (Abcam, UK), MnSOD (Cell Signaling, USA), catalase (Cell Signaling), FoxO1 (Cell Signaling), and *β*-actin (Sigma, USA).

### 2.6. Mitochondrial Membrane Potential (*ΔΨ*m) Analysis

The hPDLSC mitochondrial membrane potential was detected by a mitochondrial membrane potential assay kit with JC-1 (Beyotime) following the manufacturer's protocol. At the end of the treatment period, cells were collected in a polystyrene centrifuge tube and washed twice with wash buffer. Then, hPDLSCs were resuspended and incubated in a freshly prepared JC-1 working solution at 37°C for 20 min. Cells were analyzed using flow cytometry (Beckman, CytoFLEX) and fluorescence microscopy (ZEISS, Axio Image Z2).

### 2.7. Apoptosis Detection

The membrane phospholipid phosphatidylserine (PS) translocated to the outer leaflet of apoptotic cells. Annexin V bound to exposed PS to identify apoptotic cells. After the treatment process, the percentage of apoptotic cells was measured by a FITC Annexin V Apoptosis Detection Kit I (BD Biosciences, USA). Briefly, cells were collected after different treatments and washed twice with cold PBS. Then, the cells were stained with FITC Annexin V and 7-AAD at 25°C in the dark and analyzed by flow cytometry (Beckman, CytoFLEX).

### 2.8. Immunofluorescence Staining

hPDLSCs were seeded on sterile slides following 0 or 10 ng/mL TNF-*α* treatment. Cells were fixed in 4% formaldehyde for 10 min and permeabilized with 0.1% Triton X-100 for 10 min. After blocking with 1% BSA in PBS, the cells were incubated with primary antibody against cleaved caspase-3 (cleaved caspase-3, 1 : 400, CST) at 4°C overnight. Then, the cells were incubated with secondary antibody conjugated to Alexa 488 (Invitrogen, 1 : 400) for 1 h. Cells were incubated with Hoechst 33342 (Life Technologies) and then imaged by confocal microscopy (ZEISS, LSM780).

### 2.9. Alizarin Red Assay

An Alizarin red assay was performed to determine the level of mineralized nodule deposition. After incubation in osteogenic-inducing medium for 21 days, the cells were stained with 1% Alizarin red dye (pH 4.2, Sigma, USA) for 20 min. The cells were washed three times with PBS to remove the excess dye, and images were recorded. For quantitative analysis, 10% cetylpyridinium chloride (Sigma, USA) was added to dissolve the mineralized nodules, and the absorbance at 562 nm was measured spectrophotometrically.

### 2.10. Statistics

Data are presented as the mean ± standard error. We used one-way ANOVA to analyze the data. *p* values less than 0.05 indicated significance. Each experiment was performed at least three times.

## 3. Results

### 3.1. Antioxidant Potential of FoxO1-Overexpressing hPDLSCs

**Figure 1:**
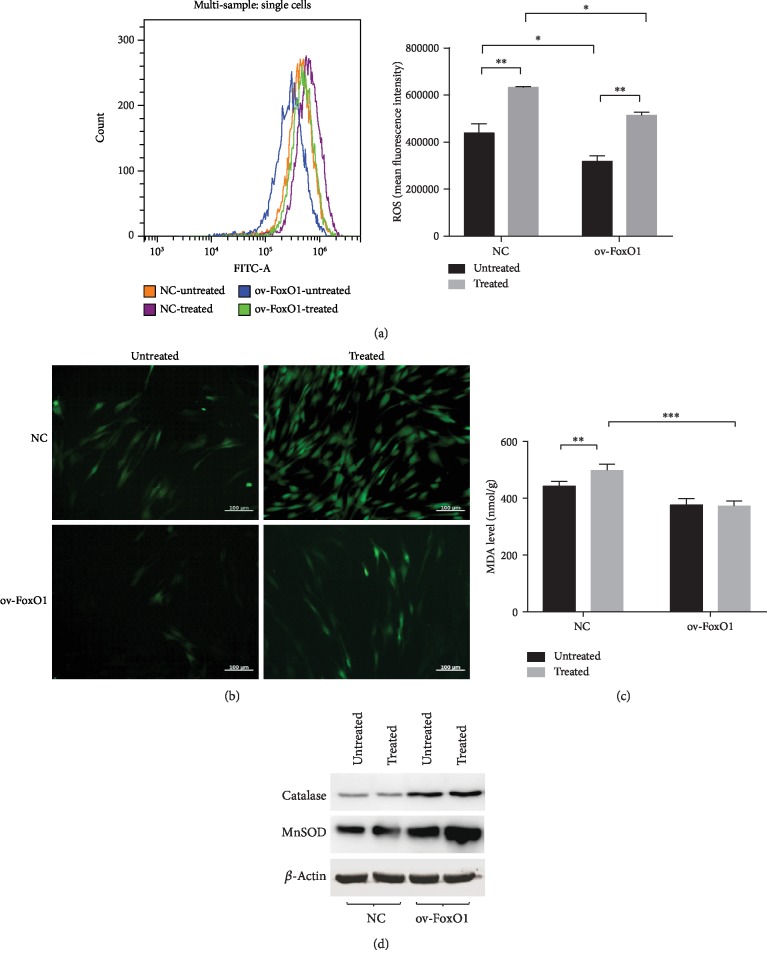
FoxO1 overexpression increased the antioxidant potential of hPDLSCs. PDLSC-pMSCV-empty (NC) and PDLSC-pMSCV-FoxO1 (ov-FoxO1) were treated with 0 or 10 ng/mL TNF-*α* for 12 h. Cellular ROS generation following different treatments was measured by staining with DCFH-DA (a, b). MDA levels in hPDLSCs were determined by a Lipid Peroxidation MDA Assay Kit following treatment with *α*-MEM or TNF-*α* (10 ng/mL) for 72 h (c). The expression of MnSOD and catalase in hPDLSCs exposed to 0 or 10 ng/mL TNF-*α* for 72 h was determined by Western blotting (d). ^∗^*p* < 0.05; ^∗∗^*p* < 0.01; ^∗∗∗^*p* < 0.001.

We identified that isolated hPDLSCs are mesenchymal stromal cells ([Supplementary-material supplementary-material-1]). In this study, 10 ng/mL TNF-*α* was employed to mimic the inflammatory niche according to a previous study [16]. First, to determine the antioxidative capacity of FoxO1 in an inflammatory environment, the ROS production of FoxO1-overexpressing hPDLSCs was detected after TNF-*α* treatment. The overexpression of FoxO1 reduced the intracellular ROS level compared with that in the control group. Stimulation with TNF-*α* increased ROS accumulation, but this ROS accumulation was reversed by FoxO1 overexpression (Figures 1(a) and 1(b)). Second, to determine whether FoxO1 overexpression exerts a protective effect on hPDLSCs under TNF-*α*-induced oxidative stress, we measured the production of MDA in hPDLSCs following different treatments. Stimulation with TNF-*α* increased the level of MDA, but FoxO1 overexpression ameliorated the change in MDA production in the presence of TNF-*α* (Figure 1(c)). Finally, to verify whether FoxO1 activates antioxidant defense, the protein levels of MnSOD and catalase were analyzed by Western blotting. FoxO1 overexpression increased the antioxidant level compared with that in the control group. Interestingly, stimulation with TNF-*α* did not remarkedly decrease the catalase and MnSOD levels as expected (Figure 1(d)). This is presumably because the TNF-*α*-induced activation of NF-*κ*B has increased antioxidant enzymes to maintain redox balance [17].

### 3.2. FoxO1 Overexpression Stabilized the Mitochondrial Membrane Potential in hPDLSCs

**Figure 2:**
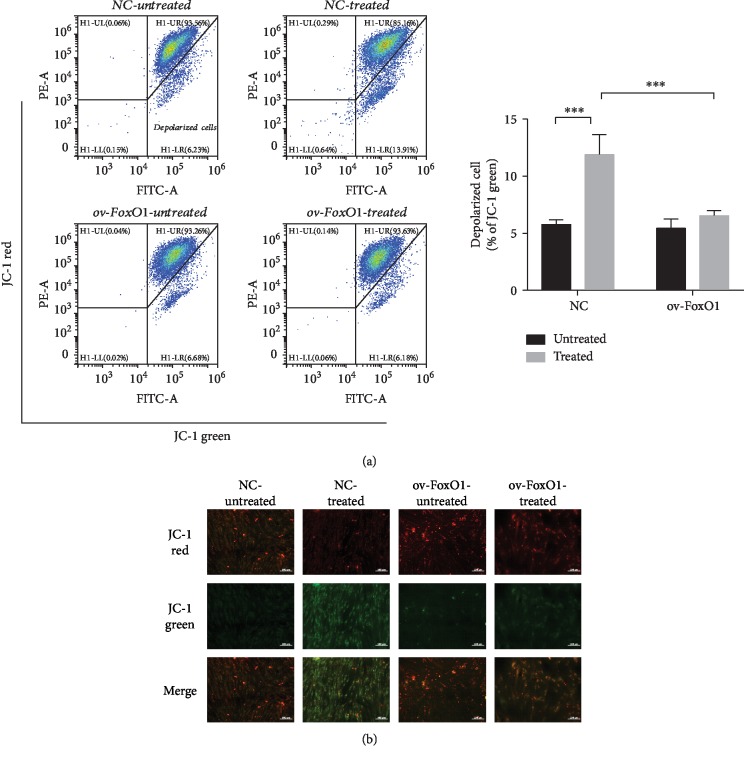
FoxO1 overexpression improved the mitochondrial membrane potential in hPDLSCs. PDLSC-pMSCV-empty (NC) and PDLSC-pMSCV-FoxO1 (ov-FoxO1) were labeled with JC-1 to determine their mitochondrial membrane potential following treatment with *α*-MEM or TNF-*α* (10 ng/mL) for 72 h (a). Quantification of depolarized cells presented as the percent of the total cells. Confocal microscopic images of hPDLSCs were labeled with JC-1 (b). JC-1 aggregates exhibited red fluorescence, and JC-1 monomers exhibited green fluorescence. ^∗∗∗^*p* < 0.001.

To evaluate the role of mitochondria in cell apoptosis and define the functional status of mitochondria under oxidative stress, we measured the mitochondrial membrane potential of hPDLSCs. Stimulation with TNF-*α* resulted in the collapse of the mitochondrial membrane potential in the control cells, but FoxO1 overexpression preserved the mitochondrial membrane potential, although the cells had been exposed to TNF-*α* (Figure 2(a)). In healthy cells with a high mitochondrial membrane potential, JC-1 forms aggregates within the mitochondria, indicated by red fluorescence. However, JC-1 remains in the cytoplasm as a monomer and exhibits green fluorescence when cells have a low mitochondrial membrane potential [18]. The formation of JC-1 aggregates was remarkably decreased, and the number of JC-1 monomers was increased in hPDLSCs exposed to TNF-*α*. However, FoxO1 overexpression reversed mitochondrial depolarization and stabilized the mitochondrial membrane potential (Figure 2(b)).

### 3.3. FoxO1 Overexpression Alleviated TNF-*α*-Stimulated Cell Apoptosis in hPDLSCs

**Figure 3:**
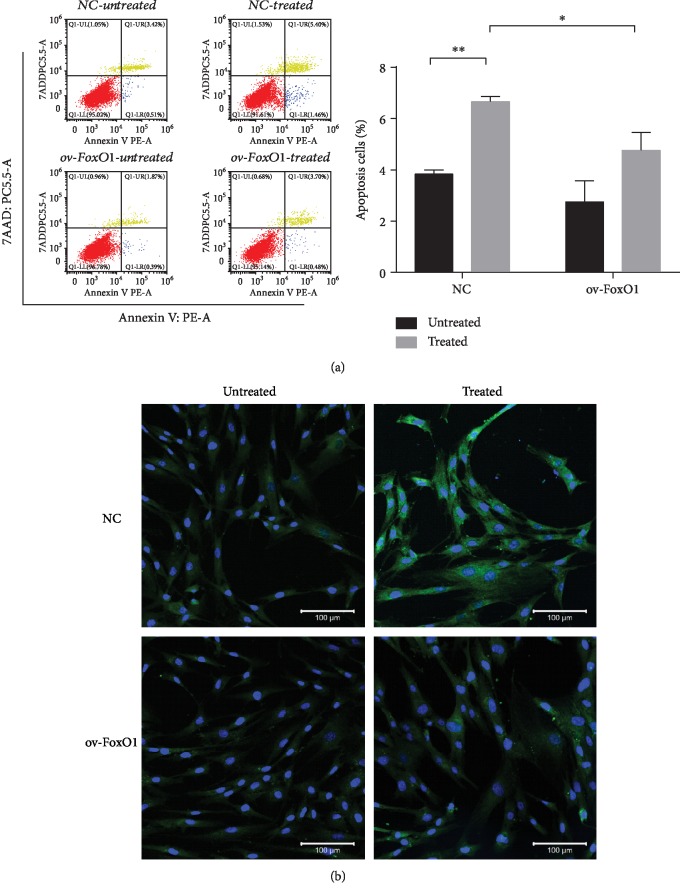
FoxO1 overexpression reduced TNF-*α*-induced apoptosis in hPDLSCs. Apoptosis was detected in PDLSC-pMSCV-empty (NC) and PDLSC-pMSCV-FoxO1 (ov-FoxO1) by Annexin V/7-AAD flow cytometry following treatment with *α*-MEM or TNF-*α* (10 ng/mL) for 72 h (a). Quantification of apoptotic cells presented as the percentage of the total cells (b). Immunofluorescence images of hPDLSCs showing cleaved caspase-3 (green) and Hoechst (blue) (c). ^∗^*p* < 0.05; ^∗∗^*p* < 0.01.

To clarify the ability of FoxO1 to prevent cell apoptosis, we evaluated cell apoptosis in hPDLSCs using FITC Annexin V/7-AAD staining. Flow cytometric analysis showed that TNF-*α* treatment increased the percentage of Annexin V/7-AAD-positive cells compared with that in the control, which was alleviated by FoxO1 overexpression (Figure 3(a)). Confocal microscopy revealed that the level of cleaved caspase-3, an apoptosis executioner, was increased in hPDLSCs treated with TNF-*α*, but FoxO1 overexpression reduced the expression of this proapoptotic factor (Figure 3(b)).

### 3.4. The Effect of FoxO1 Overexpression on the Osteoblast Differentiation of hPDLSCs Treated with TNF-*α*

**Figure 4:**
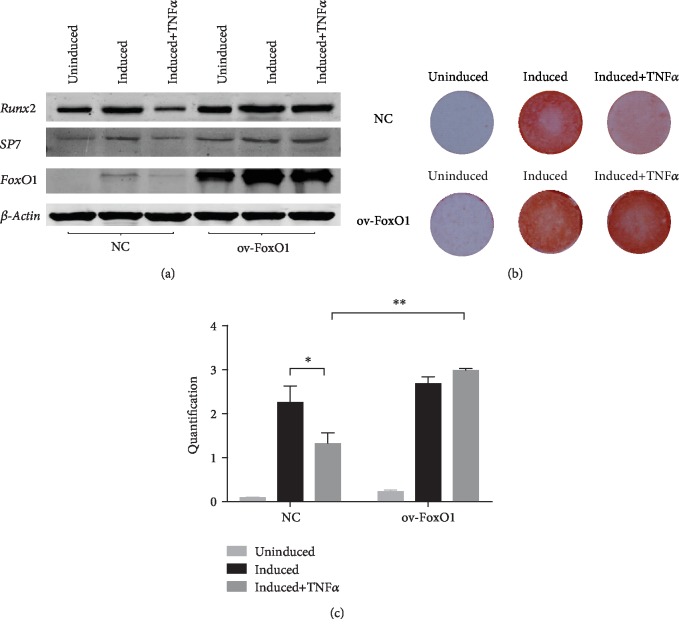
FoxO1 overexpression enhanced osteogenic differentiation in hPDLSCs. PDLSC-pMSCV-empty (NC) and PDLSC-pMSCV-FoxO1 (ov-FoxO1) were cultured in osteogenic induction medium containing 0 or 10 ng/mL TNF-*α* for 21 days. Western blot analysis of Runx2, SP7, and FoxO1 protein levels after 21 days of osteogenic induction, with *β*-actin used as a control (a). The level of mineralized nodule deposition in hPDLSCs was assessed by Alizarin red S staining (b) and quantified (c). ^∗^*p* < 0.05; ^∗∗^*p* < 0.01.

TNF-*α* was shown to depress osteogenic differentiation capacity and induce cementoblast apoptosis by activating p53 and PERK signaling [19, 20]. To determine the effect of FoxO1 on osteogenesis under inflammatory condition, we analyzed the expression of osteoblastic markers and the level of mineralized nodule deposition. TNF-*α* reduced mineralized nodule deposition and expression of the osteogenic genes Runx2 and SP7. In addition, the expression of FoxO1 declined when hPDLSCs were exposed to TNF-*α*. However, the suppressive effect of TNF-*α* on osteoblast differentiation was reversed by FoxO1 overexpression (Figure 4). Taken together, these data indicated that FoxO1 overexpression in hPDLSCs contributes to the positive regulation of osteogenesis in the inflammatory environment.

## 4. Discussion

Periodontitis is associated with an excessive inflammatory response and redox metabolism imbalance. The inflammatory response in periodontitis impairs the function of hPDLSCs, leading to the suppression of osteogenic differentiation and inhibiting tissue regeneration in periodontitis treatment. During the development of periodontitis, neutrophils release matrix metalloproteinase and proinflammatory cytokines including TNF-*α*, CCL2, and IL-6 [21, 22]. In the inflammatory environment, proinflammatory cytokines induce oxidative stress and impair the function of hPDLSCs, which severely compromises periodontal regeneration. Therefore, it is important to elevate the antioxidative ability of hPDLSCs and promote their regenerative capacity to resist the inflammatory environment and achieve satisfactory therapeutic outcomes.

FoxO1 mediates ROS generation via TXNIP-TRX and is involved in maintaining cellular redox balance [23]. Furthermore, FoxO1 resists oxidative stress by upregulating antioxidant enzymes [24, 25]. Knockdown of FoxO1 may elevate the levels of ROS because FoxO1 binding is required for MnSOD and catalase transcription activation [14, 26]. However, FoxO1 contributes to osteoblast proliferation and bone formation by promoting protein synthesis and regulating the canonical Wnt pathway [12, 15]. We observed that FoxO1 overexpression protected hPDLSCs against oxidative damage and promoted their osteogenic capacity in the inflammatory environment. Here, we shed new light on the role of FoxO1 in antioxidant defense and osteogenesis to resist adverse environments in periodontitis.

In this study, FoxO1 overexpression effectively enhanced the antioxidant potential of hPDLSCs. Overexpression of FoxO1 reduced ROS generation and elevated the expression of MnSOD and catalase. In addition, we observed that TNF-*α* simulation did not notably decrease catalase and MnSOD expression (Figure 1(d)). The slight upregulation of MnSOD might have been the consequence of TNF-*α*-induced NF-*κ*B pathway activation [17, 27]. Moreover, short-term stimulation with TNF-*α* may be incapable of impairing the antioxidant defense systems, and TNF-*α*-induced oxidative stress in hPDLSCs did not depend on decreased antioxidant levels but rather the activation of other signaling pathways. For example, TNF-*α*-induced decreases in the activities of mitochondrial complexes I, II, III, and IV and the consequent impairment of electron transfer directly produce free radicals and oxidative damage [28]. However, this study showed that overexpression of FoxO1 restrains oxidative stress. Oxidative stress reduction is accompanied by immunomodulatory effects and decreasing systemic levels of proinflammatory cytokines, preventing periodontal tissue damage [29]. The role of FoxO1 in promoting antioxidant defense suggests FoxO1 as a candidate target of periodontitis treatment.

TNF-*α* directly or indirectly causes cellular damage and tissue destruction by inducing oxidative stress, leading to lipid peroxidation, DNA damage, and protein fragmentation [30]. These effects lead to cell membrane damage, organelle injury such as mitochondrial fission, and eventual cell death. Consequently, it is important to prevent cellular damage from oxidative stress. Cell membranes contain high concentrations of polyunsaturated fatty acids. Oxygen-derived free radicals react with polyunsaturated fatty acids, leading to lipid peroxidation in cell membranes, decreased membrane fluidity, and variable membrane permeability [31, 32]. As expected, FoxO1 overexpression reduced the level of ROS and MDA production, protecting the physiology of the lipid bilayer. In addition to the peroxidation of membrane lipids, TNF-*α* causes cellular damage involving activation of the mitochondria-mediated apoptotic signaling pathway [33]. TNF-*α*-induced ROS accumulation results in the collapse of the mitochondrial membrane potential, which is important for ATP production and mitochondrial function. Loss of the mitochondrial membrane potential likely promotes mitochondrial permeability transition pore opening, leading to the release of cytochrome c from mitochondria to the cytosol and activation of the caspase cascade pathway and inducing intrinsic apoptosis [34–36]. We noticed that FoxO1 overexpression prevented hPDLSCs from undergoing apoptosis under oxidative stress by preserving the mitochondrial membrane potential and reducing caspase-3 activation. Because FoxO1 binds to the promoter regions of TXNIP and TXN, FoxO1 participates in regulating their transcription to alleviate oxidative injury and prevents apoptosis [23, 37]. In addition, FoxO1 is involved in preventing mitochondrial dysfunction and inhibiting mitochondria-mediated intrinsic apoptosis by activating JNK signaling to decrease the expression of cleaved caspase-3 and upregulate Bcl-2 expression [38, 39]. Taken together, these results suggest that the effect of FoxO1 on antioxidant defense and mitochondria protection might explain the reduced TNF-*α*-induced cell apoptosis.

TNF-*α* inhibits the osteogenic differentiation of hPDLSCs by provoking ER stress responses and the PERK pathway [20]. In addition, TNF induces mitochondrial ROS accumulation and impairs mitochondrial function to inhibit osteoblast function [40]. However, the addition of antioxidants promotes ALP activity and osteogenic markers to reverse these unfavorable phenomena [41, 42]. For instance, antioxidants such as fullerol have been reported to promote osteogenesis by elevating FoxO1 expression. The present study found that FoxO1 overexpression, which exerts an effect similar to that of antioxidants, promoted the osteogenic potential of hPDLSCs. Overexpression of FoxO1 increased mineralized nodule deposition and elevated osteogenic gene expression in the inflammatory microenvironment. FoxO1 overexpression upregulated antioxidant expression and activated antioxidant defense-facilitated osteogenic differentiation and bone formation. Thus, we hypothesize that the effect of FoxO1 on oxidative defense plays an important role in osteogenic differentiation and bone homeostasis in the inflammatory environment. In addition to facilitating protein synthesis and resisting oxidative stress to control bone mass, FoxO1 also contributes to bone formation by inhibiting the Wnt pathway [15]. In addition, FoxO1 is involved in the inflammatory process to promote bone regeneration by negatively regulating the generation of Th17 cells and IL-17 expression [43, 44]. However, more in vitro and in vivo studies are needed to confirm the role of FoxO1 in the immune system and osteogenesis to promote tissue regeneration in periodontitis.

## 5. Conclusions

The present study found that FoxO1 exerted antioxidative effort on protecting hPDLSCs from cellular oxidative damage and promoting osteogenesis in inflammatory microenvironment. It indicated that FoxO1 plays an anti-inflammatory role in periodontium regeneration in periodontitis treatment. Thus, this study contributed new insight into the anti-inflammatory capability of FoxO1 and provided new evidence for tissue regeneration in periodontitis treatment.

## Data Availability

The data used to support the findings of this study are included within the article.

## Supplementary Materials

Supplementary Materials Figure S1: characterization of PDLSCs. Single colony-forming units of hPDLSCs at 10 days (A). Immunocytochemical staining of hPDLSCs that positively expressed vimentin and negatively expressed CK18 (B). Osteoblast and adipocyte differentiation of hPDLSCs demonstrated by Alizarin red (C) and oil red O staining (D). hPDLSCs were collected and incubated with PE-labeled anti-CD34, anti-CD45, anti-CD90, and anti-CD105 and FITC-labeled anti-HLA-DR antibodies (1 : 50; BD Biosciences, USA). Expression of surface antigens on hPDLSCs was detected by flow cytometry (E).
