# Supplementary material for: FoxO1 Overexpression Ameliorates TNF-α-Induced Oxidative Damage and Promotes Osteogenesis of Human Periodontal Ligament Stem Cells via Antioxidant Defense Activation
Source: Stem Cells Int. 2019 Oct 31;2019:2120453. doi: 10.1155/2019/2120453 (PMC6875375; doi:10.1155/2019/2120453)
Supplement: Supplementary Materials — Figure S1: characterization of PDLSCs. Single colony-forming units of hPDLSCs at 10 days (A). Immunocytochemical staining of hPDLSCs that positively expressed vimentin and negatively expressed CK18 (B). Osteoblast and adipocyte differentiation of hPDLSCs demonstrated by Alizarin red (C) and oil red O staining (D). hPDLSCs were collected and incubated with PE-labeled anti-CD34, anti-CD45, anti-CD90, and anti-CD105 and FITC-labeled anti-HLA-DR antibodies (1 : 50; BD Biosciences, USA). Expression of surface antigens on hPDLSCs was detected by flow cytometry (E). [file 2120453.f1.docx]

## Supplementary Materials


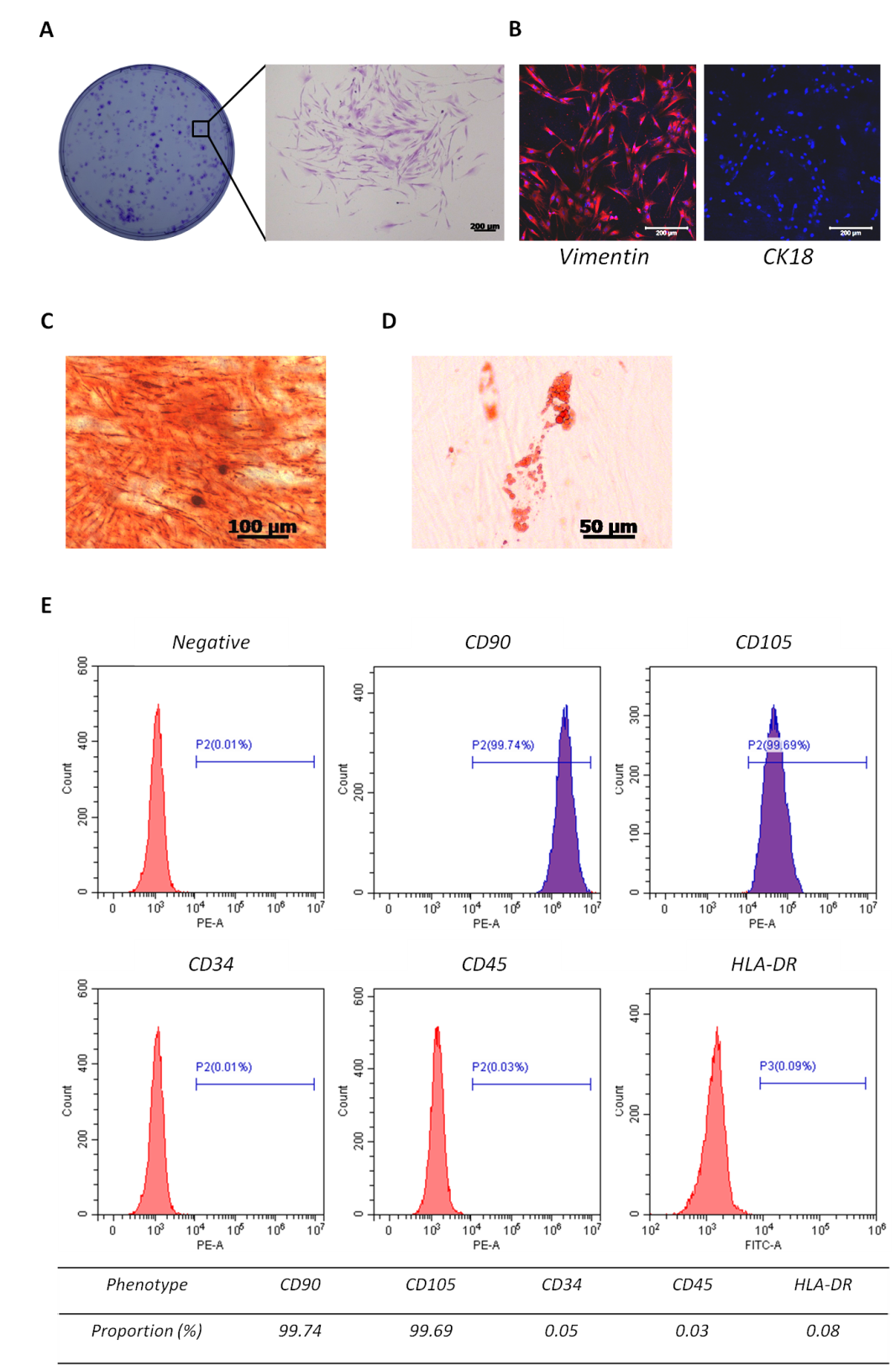


**Figure S1**: Characterization of PDLSCs. Single colony-forming units of hPDLSCs at 10 days (A). Immunocytochemical staining of hPDLSCs that positively expressed vimentin and negatively expressed CK18 (B). Osteoblast and adipocyte differentiation of hPDLSCs demonstrated by Alizarin red (C) and oil red O staining (D). hPDLSCs were collected and incubated with PE-labeled anti-CD34, anti-CD45, anti-CD90, anti-CD105 and FITC-labeled anti-HLA-DR antibodies (1:50; BD Biosciences, USA). Expression of surface antigens on hPDLSCs was detected by flow cytometry (E).
